# Use of High Frequency Ultrasound to Monitor Cervical Lymph Node Alterations in Mice

**DOI:** 10.1371/journal.pone.0100185

**Published:** 2014-06-23

**Authors:** Elyse L. Walk, Sarah McLaughlin, James Coad, Scott A. Weed

**Affiliations:** 1 Department of Neurobiology and Anatomy, West Virginia University, Morgantown, West Virginia, United States of America; 2 Department of Pathology, West Virginia University, Morgantown, West Virginia, United States of America; 3 Program in Cancer Cell Biology, West Virginia University, Morgantown, West Virginia, United States of America; 4 Animal Models and Imaging Facility, West Virginia University, Morgantown, West Virginia, United States of America; 5 Mary Babb Randolph Cancer Center, West Virginia University, Morgantown, West Virginia, United States of America; Virginia Commonwealth University, United States of America

## Abstract

Cervical lymph node evaluation by clinical ultrasound is a non-invasive procedure used in diagnosing nodal status, and when combined with fine-needle aspiration cytology (FNAC), provides an effective method to assess nodal pathologies. Development of high-frequency ultrasound (HF US) allows real-time monitoring of lymph node alterations in animal models. While HF US is frequently used in animal models of tumor biology, use of HF US for studying cervical lymph nodes alterations associated with murine models of head and neck cancer, or any other model of lymphadenopathy, is lacking. Here we utilize HF US to monitor cervical lymph nodes changes in mice following exposure to the oral cancer-inducing carcinogen 4-nitroquinoline-1-oxide (4-NQO) and in mice with systemic autoimmunity. 4-NQO induces tumors within the mouse oral cavity as early as 19 wks that recapitulate HNSCC. Monitoring of cervical (mandibular) lymph nodes by gray scale and power Doppler sonography revealed changes in lymph node size eight weeks after 4-NQO treatment, prior to tumor formation. 4-NQO causes changes in cervical node blood flow resulting from oral tumor progression. Histological evaluation indicated that the early 4-NQO induced changes in lymph node volume were due to specific hyperproliferation of T-cell enriched zones in the paracortex. We also show that HF US can be used to perform image-guided fine needle aspirate (FNA) biopsies on mice with enlarged mandibular lymph nodes due to genetic mutation of Fas ligand (Fasl). Collectively these studies indicate that HF US is an effective technique for the non-invasive study of cervical lymph node alterations in live mouse models of oral cancer and other mouse models containing cervical lymphadenopathy.

## Introduction

The most common route of dissemination for head and neck cancers is via the local lymphatic system, where patient prognosis relies heavily on the ability to detect cervical lymph node involvement [1]–[3]. Several different imaging modalities are currently used to enhance pretreatment staging of patients with head and neck squamous cell carcinoma (HNSCC), including computed tomography (CT), positron emission tomography (PET)-CT, magnetic resonance imaging (MRI) and ultrasonography [4]–[7]. Of these, ultrasound has greater clinic availability and is easiest to employ [6]. When combined with FNAC, ultrasound provides a highly accurate, sensitive and selective means to assess lymph node alterations in patients, including tumor cell metastasis [4], [6], [8].

The development of high-frequency ultrasound (HF US) technology has allowed sonography to be performed on rodent and other small animal disease models. HF US is a noninvasive, real-time technique that allows imaging of internal structures down to 30 microns using gray scale or brightness (B)-mode [9]. This resolution allows for real-time monitoring of tumor formation and progression *in vivo* in a variety of animal model systems. 3D reconstructions of HF US 2D images allows for the calculation of highly accurate tumor and lymph node volumes. In addition, power Doppler sonography is commonly used to assess and quantify blood flow velocities in tumors and lymph nodes. The combination of these two modalities is useful in quantifying tumor-induced alterations of circulatory flow [10]–[13].

Several mouse models of HNSCC have been generated that recapitulate important aspects of the human disease. These include orthotopic xenografts, genetically engineered mouse models and carcinogen-initiated tumors [14], [15]. Common carcinogens used to spontaneously generate rodent HNSCC include 7,12-dimethylbenz(a)anthracene or 9,10-dimethyl-1,2-benzanthracene (DMBA) and 4-nitroquinoline-1-oxide (4-NQO) [16]. The 4-NQO oral cancer model is a prevalent method to induce HNSCC in mice, as it closely mimics the oncogenic effect of tobacco carcinogens and copies many key molecular alterations that occur during human HNSCC development [17], [18], including lymph node metastasis [19]. Tumor induction is achieved by the addition of 4-NQO to the drinking water of immunocompetent mice, with tumor development followed over a period of several weeks to months. The degree and swiftness of carcinogenesis is dependent on the exposure time and 4-NQO dosage [20]–[22]. While many studies have investigated the effects of 4-NQO on multiple aspects of rodent oral cancer [20], [21], [23]–[28], reports examining the impact of 4-NQO exposure on murine lymph node biology are lacking [19].

While diagnostic ultrasound affords practical utility in evaluating pre- and cancerous changes within patient cervical lymph nodes, adapting HF US to evaluate cervical nodal alterations in mouse HNSCC or other model systems has not been reported. Here we show that HF US can be utilized for monitoring changes in cervical lymph nodes in 4-NQO-treated mice during the course of oral cancer progression. C57BL/6 (B6) mice treated with 4-NQO for eight weeks displayed increased lymph node volume and vascular flow prior to oral tumor development. Histological evaluation determined that pre-cancerous elevation of lymph node volume was specifically due to increased proliferation of intranodal T-cell zones. Furthermore, we show that HF US can be utilized to obtain image-guided FNA biopsy material from Fasl mice that contain chronically enlarged cervical nodes. The ability of HF US to conduct real time monitoring of murine cervical lymph node dynamics allows for the practical detection of neck node changes in mice that cannot be accomplished by conventional histology. This technique ultimately provides increased utility and accuracy for studies involving live rodent models of HNSCC and other rodent systems that model cervical node lymphadenopathy.

## Materials and Methods

### Mice

B6 and CPt.C3-Fasl^gld^/J (Fasl) mice were purchased from the Jackson Laboratory (Bar Harbor, ME). FVB mice were a generous gift from John Hollander (West Virginia University). All animal studies were approved by the WVU Institutional Animal Care and Use Committee (protocol 11-0412) and conducted in accordance with the principles and procedures outlined in the NIH Guide for the Care and Use of Animals.

### 4-NQO administration

22–24 week old B6 mice were given 50–100 µg/mL 4-NQO (Sigma, St Louis, MO) in their drinking water ad libitum for eight weeks, with water changed at weekly intervals. Normal drinking water was resumed at the end of the eight-week treatment.

### High-frequency ultrasonography and image analysis

Ultrasound imaging was performed using a VisualSonics Vevo 2100 micro-ultrasound system (Toronto, Ontario, Canada) on control (n = 4) and 4-NQO-treated (n = 3) mice. Mice were initially anesthetized with 3% isoflurane with oxygen and maintained at 1–2% isoflurane with oxygen during imaging. Anesthetized mice were positioned in dorsal recumbancy on a heated imaging platform and paws taped to electrocardiograph (ECG) leads to monitor heart and respiration rates. Body temperature was maintained at 37°C and monitored with a rectal probe thermometer. Hair was removed from the neck region using a chemical depilatory (Nair, Church & Dwight, NJ). A 40 MHz transducer was used for lower resolution overview imaging of the neck region from the thyroid gland through to the posterior tongue. All other images were acquired using a 50 MHz transducer. Images were taken in 3D-mode using combined B- and power Doppler mode. Lymph node volume and percent blood flow were determined using Vevo 2100 software after drawing regions of interest within each sequential 2D image. On average, each mouse took approximately 15 minutes to prepare and image.

### Image-guided fine needle biopsy

Enlarged mandibular cervical nodes in anesthetized Fasl mice were identified using the 50 MHz transducer focused at the lymph node center. A 27 ½ gauge needle attached to a 1 ml syringe was inserted into the micro-injector, consisting of an adjustable needle holder with micro-manipulation controls. The needle was positioned bevel side up and inserted through the skin into the mandibular node. After ∼100 µl of lymph tissue was extracted, the needle was removed and the syringe placed in a 2 µL microcentrifuge tube. The syringe was filled with 1 mL of ThinPrep media and reattached to the needle. The media was dispensed for rinsing and processed by Cytospin using a blue filter in a Thinprep 2000 processor (Cytyc, Marlborough, MA).

### Immunohistochemical analysis

Whole necks, cervical lymph nodes and tongues were dissected, rinsed in PBS, fixed in 10% neutral buffered formalin (Fisher, Pittsburgh, PA) and embedded in paraffin. Whole neck sections required decalcification using Rapid-Cal•Immuno decalcification solution (BBC Biochemical, Seattle, WA) after fixation. Five-micrometer sections from tissue blocks were stained with hematoxylin and eosin (H&E) or immunolabeled with prediluted cytokeratin 14 antibody (Abcam, Cambridge, MA) using a Discovery XT automated staining system (Ventana Medical Systems, Tucson, AZ). Lymph node paracortical T-cell zone expansion analysis was performed by pathological evaluation on H&E stained sections after grouping nodes by right or left side. Individual nodes were scored as having none, moderate or robust enlargement. Histological images were obtained using an Olympus AX70 Provis microscope (Center Valley, PA).

### Statistical analysis

Differences between groups were evaluated using Student's *t*-test with significance determined at p≤0.05.

## Results

### High-frequency ultrasound detection of mouse cervical lymph nodes

**Figure 1:**
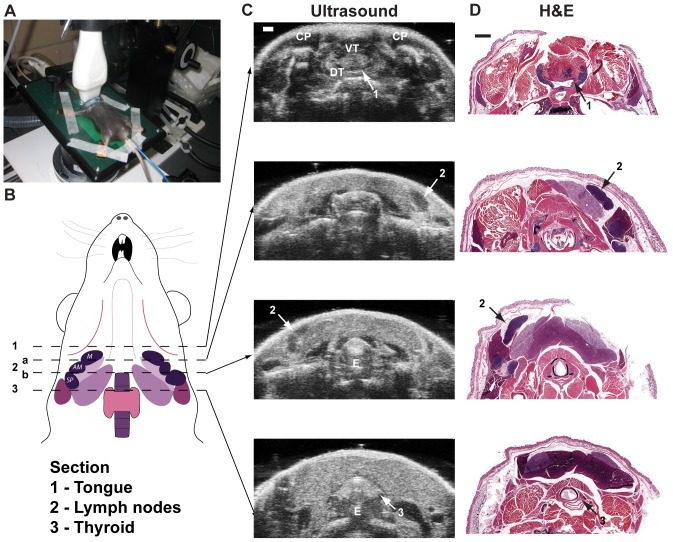
Mapping of mouse cervical lymph nodes by high frequency ultrasound. **A.** Overview image of the HF US platform for cervical lymph node evaluation. An anesthetized B6 mouse is shown positioned on the Vevo 2100 heated imaging platform with the ventral side exposed. Each paw is tapped to a monitoring electrode and the rectal probe (blue) secured to the stage. The transducer (white) is positioned over the ventral neck area. **B.** Diagram showing relative locations of murine cervical lymph nodes. Individual neck sections visualized by HF US imaging and histology are indicated by dashed lines. Arrows denote specific positions of each mapped section relative to corresponding ultrasound and histology images. Each imaged anatomical location is numbered. M, mandibular node. AM, accessory mandibular node. SP, superficial parotid node. **C.** Serial transverse sections of the mouse neck imaged by HF US corresponding to the indicated anatomic regions in (B). **D.** Transverse cervical H&E stained histological sections corresponding to the HF US sections in (C). Arrows labeled “2” denote mandibular node as diagrammed in B. Scale bar  = 1 mm. CP, cheek pouch. VT, ventral tongue. DT, dorsal tongue. E, esophagus.

Cervical ultrasound is a commonly utilized tool for non-invasive imaging of lymph nodes in the patient neck, where it is frequently combined with MRI and PET/CT to determine patient staging in HNSCC and other diseases. While several publications describe the features of benign and malignant cervical lymph nodes in humans [29]–[33], studies detailing the suitability and use of US to image normal or diseased mouse cervical nodes are lacking. We initially conducted HF US on untreated B6 mice to identify and map the three supraclavicular (mandibular, accessory mandibular and superficial parotid [34]) cervical lymph nodes in the murine neck that drain the oral cavity tissues. Anesthetized mice were imaged on a heated platform with the Vevo 3D-mode scanner in order to automate the process (Figure 1A). For point of reference, the transducer was first focused on the thyroid gland, where it is well-defined as a hyperechoic solid structure when imaged by gray scale sonography (Figure 1B and C) [35]–[38]. Subsequent serial HF US images were taken of the entire neck region, starting at the jaw base and moving proximally to the thyroid (diagramed in Figure 1B). Corresponding HF US images identified each cervical node as hypoechoic oval structures within dense hyperechoic regions (Figure 1C). These hyperechoic areas primarily contain adipose, salivary gland and skeletal muscle tissue adjacent to the cervical lymph nodes, with the nodes positioned just below the integument when identified by histological analysis of parallel tissue sections (Figure 1D). The superficial nature of the cervical nodes increased their mobility due to the pressure placed by the transducer on the neck, resulting in equivalent right and left nodes appearing in different imaging planes. This was countered by making compensatory adjustments to the imaging stand while maintaining the transducer in a stationary position. Insertion of a metal feeder needle into the oral cavity during imaging ablated the ultrasound signal, allowing additional imaging of buccal regions and esophagus to provide a more comprehensive sonographic depiction of the murine oral cavity (Figure S1).

**Figure 2:**
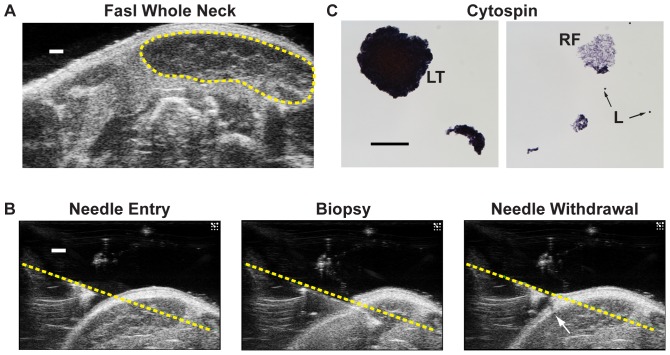
Image-guided fine needle biopsy of Fasl mandibular lymph nodes. **A.** Transverse section of a Fasl mouse neck imaged with HF US. The enlarged cervical mandibular node is evident as an oval hypoechoic region near the skin surface (circumscribed in yellow). Scale bar  = 1 mm. **B.** Frames from fine needle biopsy of a Fasl mandibular node guided by HF US. Images show the position of the sampling hyperechoic needle tip prior to cervical skin penetration (*left*), position of the needle during tissue removal (*middle*), and following needle withdrawal (*right*). Note the break in the skin following needle withdrawal (arrow). The angle and trajectory of the dorsal needle surface is denoted by the yellow dotted line. Scale bar  = 1 mm. The entire procedure is shown in Video S1. **C.** Examples of lymph tissue obtained by HF US guided FNA of a Fasl cervical mandibular node following staining and processing by cytospin. Scale bar  = 100 µm. LT; lymph tissue, RF; reticular fibers, L; individual lymphocytes.

Since FNAC of lymph nodes is used to determine patient tumor staging and for other diagnostic purposes, we determined the feasibility of conducting HF US image-guided FNAC analysis on live mice. The cervical nodes in B6 mice proved too small and mobile obtain a FNA. We therefore used Fasl mice that contain enlarged lymph nodes due to systemic autoimmunity [39], [40] that mimic the size of human lymph nodes (Figure 2A). FNAC of Fasl cervical mandibular nodes was performed using an image-guided microinjection system with the 50 MHz transducer focused at the presumed lymph node center (Figure 2B; Video S1). The entire biopsy can be seen in Video S1. Analysis of the aspirated material following Cytospin concentration exclusively revealed cellular and extracellular lymph node components, including large clumps of lymph tissue, individual lymphocytes and reticular fibers (Figure 2C). These results indicate that HF US can be successfully adapted for FNA analysis on enlarged cervical lymph nodes in mice.

### 4-NQO induces pre-cancerous enlargement of mouse submandibular lymph nodes

**Figure 3:**
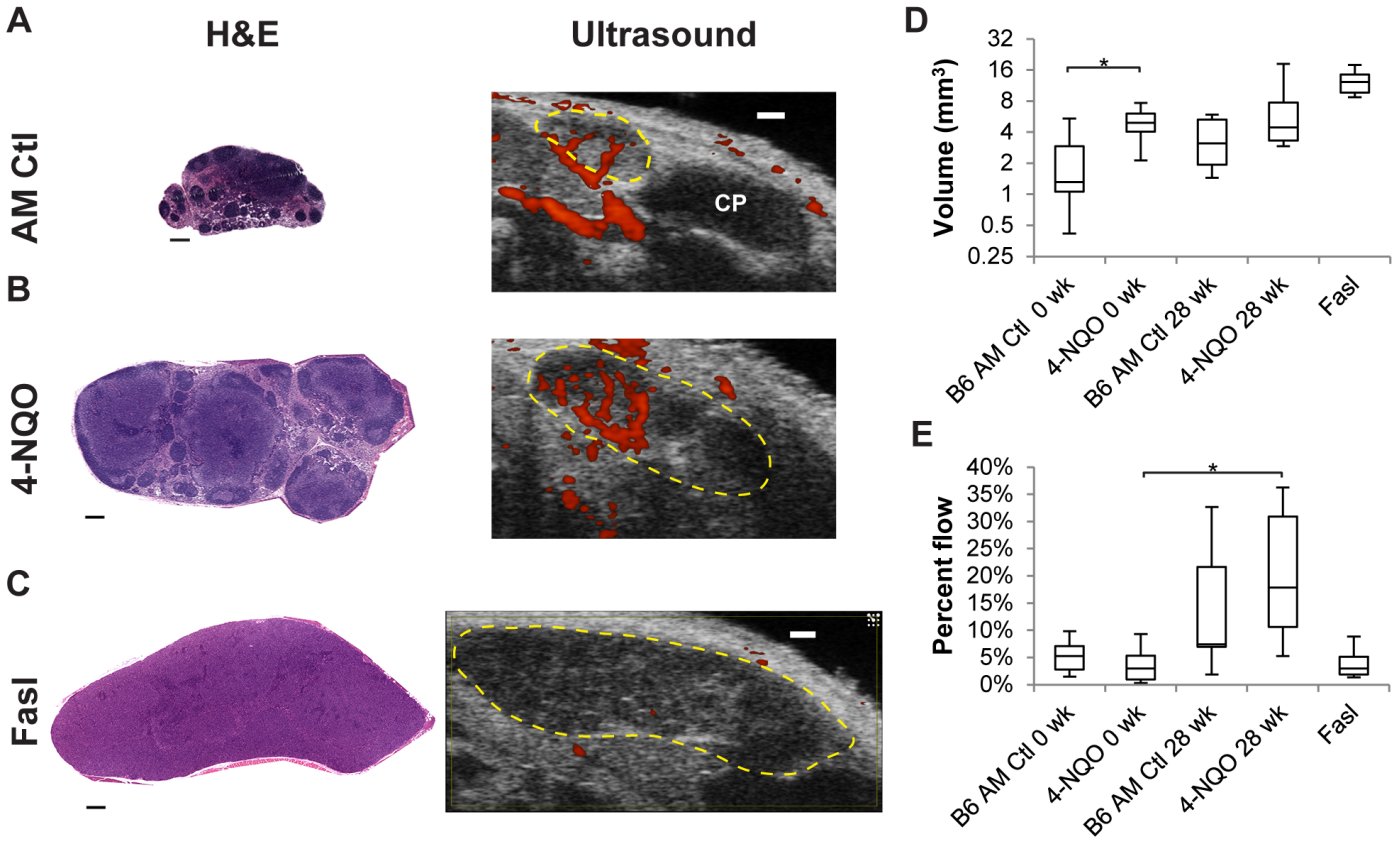
4-NQO exposure induces precancerous alterations in mouse mandibular lymph nodes. **A–C.** Images of dissected H&E and whole animal HF US (ultrasound) mandibular lymph nodes from representative age-matched (AM) control (**A**), 4-NQO-treated (28 wk) (**B**) and Fasl (**C**) mice. Lymph node borders in the HF US images are indicated in yellow. Vascular flow identified by power Doppler imaging is shown in red. Power Doppler flow dynamics for each condition are visualized in Video S2–S4. H&E scale bar  = 250 µm, ultrasound scale bar  = 1 mm. CP, Cheek Pouch. **D&E.** Analysis of lymph nodes by HF US. 4-NQO treated mice at 0 and 28 wk were imaged after 8 week 4-NQO treatment and study end point. B6 age-matched (AM) Ctl 0 and 28 wk mice were imaged at the same age as 4-NQO treated mice. The Fasl lymph node data is included for comparison. **D.** 4-NQO exposure induces increased mandibular lymph node volume. **E.** 4-NQO exposure increases vascular flow in mandibular nodes. N = 6 lymph nodes from 3 mice per group, except for the controls, where N = 8 lymph nodes were analyzed from 4 mice. Box and whisker plots show minimum, 25^th^, median, 75^th^ and maximum values, respectively. *, p≤0.05.

We next used HF US to monitor cervical lymph node changes in mice following oral 4-NQO exposure designed to induce tumorigenesis. B6 mice were selected since this strain exhibits near complete penetrance and predictable oral tumor course in response to 4-NQO treatment [28], [29]. Mice were given 4-NQO continuously for eight weeks as previously reported [20]–[22]. For clarity, the end of the eight week treatment period is denoted as the zero week time point in the study. Mice formed oral lesions similar to those reported in previous studies, starting as early as 19 weeks post-treatment [20], [22] (Figure S2A). The neck region in control and 4-NQO treated mice was imaged every four weeks by HF US for an additional 28 weeks after the zero week time point. B-mode imaging of the mandibular and accessory mandibular lymph nodes in 4-NQO treated mice at the end of the entire 36 week study period indicated slight increases in lymph node size compared to nodes from age-matched control animals (Figure 3A and B, Videos S2-S3). In a separate study, 4-NQO-treated B6 mice developed lymph node metastasis by 33 weeks post-4-NQO treatment (41 weeks total), demonstrating that 4-NQO-induced tumors are capable of undergoing lymph node metastasis during the later stages of oral cancer progression (Figure S2B).

The increased mandibular node size in 4-NQO treated mice at the end of 36 weeks was comparable to the mandibular node size in Fasl mice in many instances (Figure 3C, Video S4). Three dimensional volume measurements revealed median mandibular nodal volumes of 3.1 mm^3^ in control mice and 4.4 mm^3^ in 4-NQO treated mice 28 weeks after cessation of 4-NQO treatment (Figure 3D). Interestingly, mandibular node volume measured by HF US at the end of the initial eight-week 4-NQO treatment period (prior to tumor onset) was significantly greater than nodes in control mice (Figure 3D; 1.3 mm^3^ vs 4.9 mm^3^, respectively). This finding was surprising since there was no evidence of tumor onset in 4-NQO treated mice at this time, suggesting an early inflammatory response in these nodes, potentially due to hyperkeratosis present on the tongues of these animals (Figure S2A, 0 weeks post-4-NQO). Power Doppler analysis of the mandibular nodes indicated that while intranodal median vascular flow rates were comparable within nodes in age-matched control and 4-NQO treated mice immediately following 4-NQO treatment, median vascular flow in 4-NQO exposed nodes was increased by close to 15% in mice 28 weeks after the end of 4-NQO exposure (Figure 3E). Although median blood flow percentages were largely different between age-matched control and 4-NQO treated mice at 28 weeks (7.4% vs 17.83%, Figure 3E), the overall change did not reach statistical significance. Blood flow between age-matched non-treated controls at 0 and 28 weeks also did not increase significantly. The increased nodal blood flow in 28 wk animals was not due to the increase in lymph node volume in these mice, since the amount of vascular flow in Fasl mandibular nodes was comparable to the flow percentages in control and 4-NQO treated mice at the zero week time point (Figure 3E). Collectively these results suggest that 4-NQO treatment in B6 mice results in precancerous mandibular lymph node enlargement accompanied by increased intranodal blood flow during tumor onset and progression.

### Acute 4-NQO exposure in B6 mice enhances expansion of the mandibular lymph node paracortical/T-cell zone

**Figure 4:**
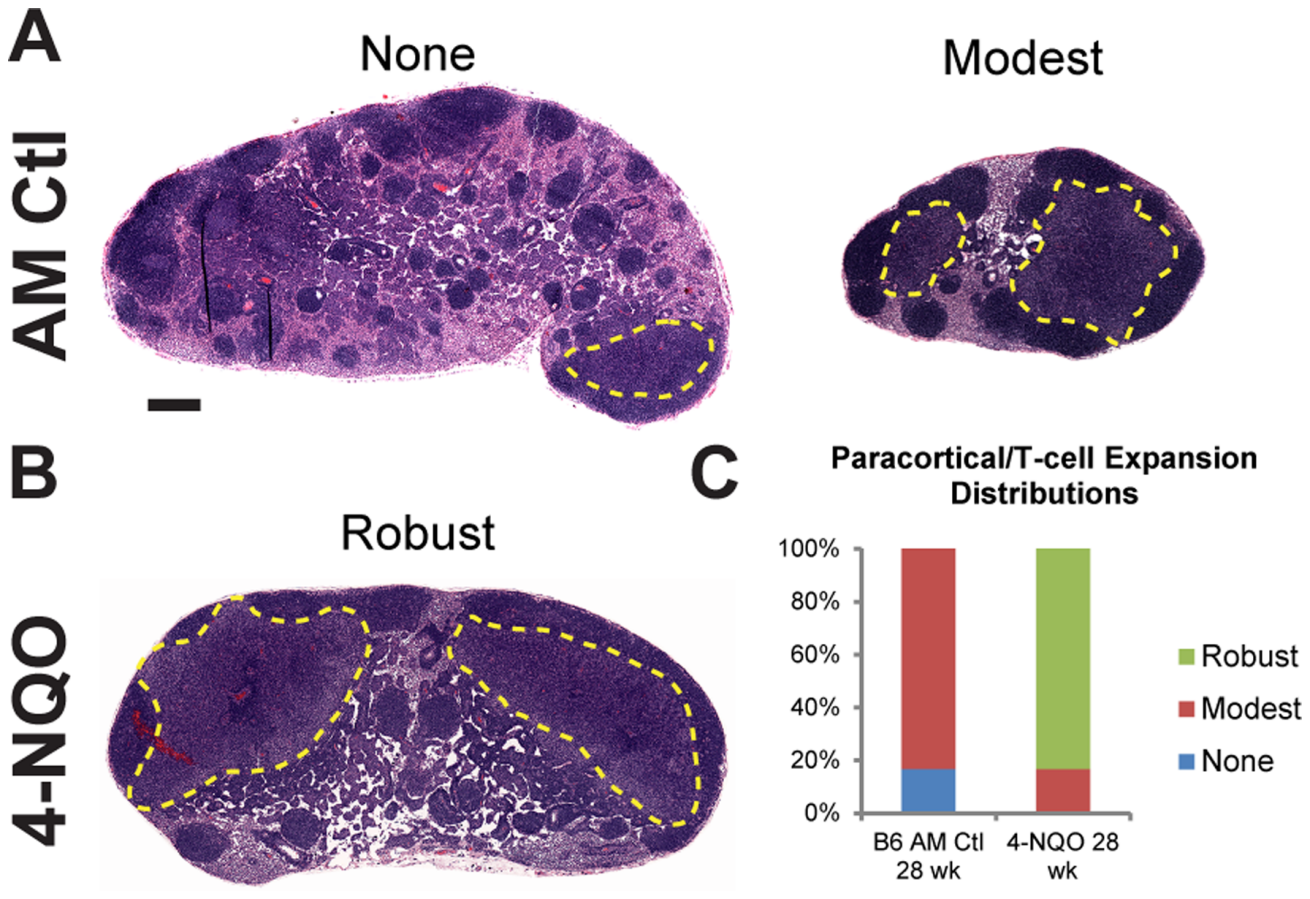
4-NQO treatment induces paracortical/T-cell zone hyperplasia in mandibular lymph nodes. Representative examples of H&E stained, dissected mandibular lymph nodes from age-matched (**A**) control and (**B**) 4-NQO-treated (28 wk) mice. T-cell zone expansions in each node are circumscribed in yellow. Scale bar  = 250 µm. **C.** Distribution of nodal paracortical/T-cell zone hyperplasia. Mandibular lymph nodes were pathologically scored and grouped according to relative scale of T-cell zone involvement, using the following scale: None, absent to focal limited expansion; Modest, multifocal or focal up to moderate expansion; Robust, multifocal moderate expansion and/or confluence of paracortical subregions.

Mandibular lymph nodes from 4-NQO exposed mice 28 weeks post-treatment displayed areas of increased lymphocyte density compared to age-matched controls (Figure 3B versus Figure 3A), suggesting that lymphocyte proliferation could be responsible for the increased submandibular nodal volume observed by HF US. Pathological evaluation of H&E-stained sections containing all cervical lymph nodes from both sides of the neck revealed varying degrees of enlargement of the T-cell enriched paracortical zones in control and 4-NQO exposed mice (Figure 4A and 4B). However, lymph nodes in 4-NQO exposed mice had a greater degree of paracortical T-cell enlargement, with over 80% of the nodes scored as containing robust expansion of this region (Figure 4C). In contrast, none of the paracortical regions in control nodes displayed more than moderate T-cell expansion (Figure 4C). These results suggest that 4-NQO exposure induces increased cervical lymph node volume attributable to specific hyperproliferation of the nodal T-cell population.

## Discussion

Clinical sonography has emerged as an important means of monitoring cervical lymph node changes in HNSCC and other oral diseases. In this study, we show that cervical lymph nodes in mouse can be effectively identified and imaged by HF US. Combined gray scale and power Doppler sonography revealed increased nodal volume and blood flow in mice treated with 4-NQO prior to tumor formation. The increase in overall pre-cancerous cervical node size in 4-NQO exposed mice can be attributed to expansion of the paracortical/T-cell zone within the node. We also demonstrate that HF US image-guided biopsies can be successfully conducted on live mice using a mutant Fasl strain that displays chronic cervical lymphadenopathy. Collectively these results support the application and utility of HF US for the minimally invasive study of cervical lymph nodes in mouse models of HNSCC and other diseases.

Examination of the mouse cervical region by HF US provides an in vivo map of cervical lymph node position that closely matches node location in histological sections. This mapping is in agreement with previous studies of the thyroid and tongue that charted these regions on a more limited level [36], [41]. Due to their inherent small size, an accurate, comprehensive in situ portrayal of mouse cervical lymph nodes is useful not only to mouse models of HNSCC, but other systems pertaining to illness causing cervical lymphadenopathy, including thyroid cancer [37], [38] and bacterial infection [42]. HF US can also be applied to study cervical organs other than lymph nodes. Salivary gland diseases such as salivary gland tumors, sialolithiasis, sialodenitis and Sjogren's syndrome [43] all have the potential to have organ-induced alterations visualized by HF US in rodent models.

FNAC is an important technique used to aid in diagnosing nodal involvement in HNSCC and other diseases. Here we demonstrate that image-guided FNAC can be successfully utilized to obtain biopsy material from Fasl mice with systemic lymphadenopathy. These mice have lymph node sizes similar to humans [44], [45], with volumes comparable to enlarged cervical nodes due to 4-NQO exposure and subsequent HNSCC tumor progression (Figure 3D). This implies that successful biopsies can be performed on any enlarged mouse lymph with similar volume. HF US-guided FNAC therefore has the potential to detect lymph node metastases in HNSCC mouse models, and is planned for future studies where longer term HF US monitoring of animals from pre-cancerous stages through tumor development and progression is achieved. The ability to conduct image-guided FNAC on cervical lymph nodes imparts translational impact on such mouse model studies, where tumor staging is typically based on the degree of nodal metastases [46], [47].

Mice orally treated with 4-NQO displayed several different changes in cervical lymph node biology unrelated to metastatic involvement that can be overlooked if not for the real-time capabilities of ultrasonography. 4-NQO-treated mice develop enlarged lymph nodes that correspond with early alterations to the tongue epithelium, where enlargement is maintained during tumor onset and progression. While enlarged nodes can be expected in response to neoplastic development, growth and/or metastasis, our findings indicate that significant lymph node enlargement occurs before tumor formation occurs in the oral cavity. The increased node size can be explained by paracortical/T-cell zone hyperplasia within the cervical nodes (Figure 4). Similar findings of paracorticial T-cell expansion have been observed in patients with oral cavity or oropharynx tumors [48], [49], but the underlying mechanism for this is unclear. Patient intranodal paracortical expansion is more pronounced in nodes without tumor infiltration [48], [49], in agreement with our data, as we did not observe metastasis in any analyzed cervical lymph node in this study (Figure 4). However, since 4-NQO-treated mice develop cervical node metastases at time points later than what were monitored in the present study (Figure S2B), it is conceivable that HF US can be employed to analyze volume changes in cervical nodes containing tumor metastases, as noted above.

In addition to increased cervical lymph node volume, power Doppler HF US demonstrated that 4-NQO treated mice have greater median blood flow in their mandibular node after tumor onset (Figure 3E). Primary tumors have been shown to induce vasculature reorganization within downstream lymph nodes, preparing the nodal microenvironment (“soil”) prior to tumor cell arrival (“seed”) in order to better support metastatic colonization [50]. This is achieved by angiogenic induction of microvasculature, including high endothelial venules, within lymph nodes before tumor cell arrival [50]–[52]. However, we did not observe changes in cervical node microvessel density or size in 4-NQO treated mice (data not shown). The systemic cause for the 4-NQO-mediated increase in cervical nodal blood flow is currently under investigation.

In summary, we demonstrate the benefits of using HF US technology to monitor cervical lymph node alterations in a mouse model of oral cancer. Real-time monitoring of lymph node biological responses is an important aspect in therapeutic and biomarker development. Detection of lymph node metastasis without the need for immediate sacrifice allows for more comprehensive and long-term study of animal disease models. In addition to HNSCC, other disease models that induce murine cervical lymphadenopathy may benefit from the application of HF US, allowing improved evaluation of cervical nodes in a variety of experimental prognostic and diagnostic settings.

## Supporting Information

Figure S1 **Visualization of regions within the mouse neck by high-frequency ultrasound.** HF US imaging of the mouse oral cavity following placement of an oral gavage needle in mouth to dampen the US signal, aiding with identification of different sections during imaging. Arrows point to area probed with needle, which can be seen in each image blocking ultrasound signal. Scale bar  = 1 mm.
(TIF)

Figure S2 **4-NQO exposure induces changes in mouse tongue epithelium similar to human HNSCC and results in cervical lymph node metastasis.** **A.** H&E and cytokeratin 14 staining of representative mouse tongues: control untreated, after 8 weeks of treatment, and after termination at 19 weeks due to tumor burden as an example to validate the ability of 4-NQO to induce oral tumors. Scale bar  = 100 µm. **B**. Cytokeratin 14 staining of mouse mandibular node from 4-NQO-treated animal 33 weeks after the end of 4-NQO treatment. Inset demonstrates cytokeratin 14-positive cells indicating epithelial origin, confirming tumor metastasis.
(TIF)

Video S1 **Image-guided fine needle biopsy of Fasl mouse lymph node.**
(AVI)

Video S2 **HF US and Power Doppler of age-matched control mouse cervical lymph node.** Representative video of 3D scan of control mandibular node using combined B-mode and power Doppler imaging modalities. The red areas within the video represent blood flow.
(AVI)

Video S3 **HF US and Power Doppler of 4-NQO-treated (28 wk) mouse cervical lymph node.** Representative video of 3D scan of 4-NQO-treated mandibular node using combined B-mode and power Doppler imaging modalities. The red areas within the video represent blood flow.
(AVI)

Video S4 **HF US and Power Doppler of Fasl mouse cervical lymph node.** Representative video of 3D scan of Fasl mandibular node using combined B-mode and power Doppler imaging modalities. The red areas within the video represent blood flow.
(AVI)

## References

[1] Noguti J, De Moura CF, De Jesus GP, Da Silva VH, Hossaka TA, et al (2012) Metastasis from oral cancer: An overview. Cancer Genomics-Proteomics 9: 329–335. 22990112

[2] Argiris A, Karamouzis MV, Raben D, Ferris RL (2008) Head and neck cancer. The Lancet 371: 1695–1709. 10.1016/S0140-6736(08)60728-X PMC7720415 18486742

[3] Leemans CR, Tiwari R, Nauta JJ, van der Waal I, Snow GB (1993) Regional lymph node involvement and its significance in the development of distant metastases in head and neck carcinoma. Cancer 71: 452–456. 8422638 10.1002/1097-0142(19930115)71:2<452::aid-cncr2820710228>3.0.co;2-b

[4] De Bondt R, Nelemans P, Hofman P, Casselman J, Kremer B, et al (2007) Detection of lymph node metastases in head and neck cancer: A meta-analysis comparing US, USgFNAC, CT and MR imaging. Eur J Radiol 64: 266–272. 17391885 10.1016/j.ejrad.2007.02.037

[5] Liao L, Lo W, Hsu W, Wang C, Lai M (2012) Detection of cervical lymph node metastasis in head and neck cancer patients with clinically N0 neck—a meta-analysis comparing different imaging modalities. BMC Cancer 12: 236. 22691269 10.1186/1471-2407-12-236 PMC3476985

[6] Ng S, Ko S, Toh C, Chen Y (2006) Imaging of neck metastases. Chang Gung Med J 29: 119. 16767959

[7] Van den Brekel, Michiel WM, Castelijns JA, Snow GB (1994) Detection of lymph node metastases in the neck: Radiologic criteria. RADIOLOGY-OAK BROOK IL- 192: 617–617. 10.1148/radiology.192.3.8058923 8058923

[8] Stoeckli SJ, Haerle SK, Strobel K, Haile SR, Hany TF, et al (2012) Initial staging of the neck in head and neck squamous cell carcinoma: A comparison of CT, PET/CT, and ultrasound-guided fine-needle aspiration cytology. Head Neck 34: 469–476. 21604319 10.1002/hed.21764

[9] Greco A, Mancini M, Gargiulo S, Gramanzini M, Claudio P, et al. (2011) Ultrasound biomicroscopy in small animal research: Applications in molecular and preclinical imaging. Journal of Biomedicine and Biotechnology 2012. 10.1155/2012/519238 PMC3202139 22163379

[10] Jugold M, Palmowski M, Huppert J, Woenne EC, Mueller MM, et al (2008) Volumetric high-frequency doppler ultrasound enables the assessment of early antiangiogenic therapy effects on tumor xenografts in nude mice. Eur Radiol 18: 753–758. 18084768 10.1007/s00330-007-0825-5

[11] Kodama T, Tomita N, Yagishita Y, Horie S, Funamoto K, et al (2011) Volumetric and angiogenic evaluation of antitumor effects with acoustic liposome and high-frequency ultrasound. Cancer Res 71: 6957–6964. 21983036 10.1158/0008-5472.CAN-11-2389

[12] Loveless ME, Li X, Huamani J, Lyshchik A, Dawant B, et al (2008) A method for assessing the microvasculature in a murine tumor model using contrast-enhanced ultrasonography. Journal of Ultrasound in Medicine 27: 1699–1709. 19022995 10.7863/jum.2008.27.12.1699 PMC2649799

[13] Snyder CS, Kaushal S, Kono Y, Cao HST, Hoffman RM, et al (2009) Complementarity of ultrasound and fluorescence imaging in an orthotopic mouse model of pancreatic cancer. BMC Cancer 9: 106. 19351417 10.1186/1471-2407-9-106 PMC2679761

[14] Sano D, Myers JN (2009) Xenograft models of head and neck cancers. Head Neck Oncol 1: 32–3284-1-32 10.1186/1758-3284-1-32; 10.1186/1758-3284-1-32. 19678942 10.1186/1758-3284-1-32 PMC2737672

[15] Kim S (2009) Animal models of cancer in the head and neck region. Clinical and experimental otorhinolaryngology 2: 55–60. 19565028 10.3342/ceo.2009.2.2.55 PMC2702728

[16] Lu S, Herrington H, Wang X (2006) Mouse models for human head and neck squamous cell carcinomas. Head Neck 28: 945–954. 16721744 10.1002/hed.20397

[17] Kanojia D, Vaidya MM (2006) 4-nitroquinoline-1-oxide induced experimental oral carcinogenesis. Oral Oncol 42: 655–667. 16448841 10.1016/j.oraloncology.2005.10.013

[18] Vitale-Cross L, Czerninski R, Amornphimoltham P, Patel V, Molinolo AA, et al (2009) Chemical carcinogenesis models for evaluating molecular-targeted prevention and treatment of oral cancer. Cancer Prevention Research 2: 419–422. 19401522 10.1158/1940-6207.CAPR-09-0058

[19] Li J, Liang F, Yu D, Qing H, Yang Y (2012) Development of a 4-nitroquinoline-1-oxide model of lymph node metastasis in oral squamous cell carcinoma. Oral Oncol. 10.1016/j.oraloncology.2012.10.013 23187306

[20] Hasina R, Martin LE, Kasza K, Jones CL, Jalil A, et al (2009) ABT-510 is an effective chemopreventive agent in the mouse 4-nitroquinoline 1-oxide model of oral carcinogenesis. Cancer Prevention Research 2: 385–393. 19336725 10.1158/1940-6207.CAPR-08-0211 PMC2702843

[21] Tang XH, Knudsen B, Bemis D, Tickoo S, Gudas LJ (2004) Oral cavity and esophageal carcinogenesis modeled in carcinogen-treated mice. Clin Cancer Res 10: 301–313. 14734483 10.1158/1078-0432.ccr-0999-3

[22] Vitale-Cross L, Molinolo AA, Martin D, Younis RH, Maruyama T, et al (2012) Metformin prevents the development of oral squamous cell carcinomas from carcinogen-induced premalignant lesions. Cancer Prevention Research 5: 562–573. 22467081 10.1158/1940-6207.CAPR-11-0502 PMC3429367

[23] Czerninski R, Amornphimoltham P, Patel V, Molinolo AA, Gutkind JS (2009) Targeting mammalian target of rapamycin by rapamycin prevents tumor progression in an oral-specific chemical carcinogenesis model. Cancer Prevention Research 2: 27–36. 19139015 10.1158/1940-6207.CAPR-08-0147

[24] Zhou G, Hasina R, Wroblewski K, Mankame TP, Doçi CL, et al (2010) Dual inhibition of vascular endothelial growth factor receptor and epidermal growth factor receptor is an effective chemopreventive strategy in the mouse 4-NQO model of oral carcinogenesis. Cancer Prevention Research 3: 1493–1502. 20978113 10.1158/1940-6207.CAPR-10-0135 PMC3408865

[25] Vered M, Allon I, Buchner A, Dayan D (2007) Stromal myofibroblasts and malignant transformation in a 4NQO rat tongue carcinogenesis model. Oral Oncol 43: 999–1006. 17257886 10.1016/j.oraloncology.2006.11.007

[26] Wilkey JF, Buchberger G, Saucier K, Patel SM, Eisenberg E, et al (2009) Cyclin D1 overexpression increases susceptibility to 4-nitroquinoline-1-oxide-induced dysplasia and neoplasia in murine squamous oral epithelium. Mol Carcinog 48: 853–861. 19263437 10.1002/mc.20531 PMC2736315

[27] Leeman-Neill RJ, Seethala RR, Singh SV, Freilino ML, Bednash JS, et al (2011) Inhibition of EGFR-STAT3 signaling with erlotinib prevents carcinogenesis in a chemically-induced mouse model of oral squamous cell carcinoma. Cancer Prevention Research 4: 230–237. 21163936 10.1158/1940-6207.CAPR-10-0249 PMC3076320

[28] Yuan B, Oechsli MN, Hendler FJ (1997) A region within murine chromosome 7F4, syntenic to the human 11q13 amplicon, is frequently amplified in 4NQO-induced oral cavity tumors. Oncogene 15: 1161–1170 10.1038/sj.onc.1201269. 9294609 10.1038/sj.onc.1201269

[29] Chang DB, Yuan A, Yu CJ, Luh KT, Kuo SH, et al (1994) Differentiation of benign and malignant cervical lymph nodes with color doppler sonography. Am J Roentgenol 162: 965–968. 8141027 10.2214/ajr.162.4.8141027

[30] Ahuja A, Ying M, Ho S, Antonio G, Lee Y, et al (2008) Ultrasound of malignant cervical lymph nodes. Cancer Imaging 8: 48. 18390388 10.1102/1470-7330.2008.0006 PMC2324368

[31] Rubaltelli L, Khadivi Y, Tregnaghi A, Stramare R, Ferro F, et al (2004) Evaluation of lymph node perfusion using continuous mode harmonic ultrasonography with a second-generation contrast agent. Journal of ultrasound in medicine 23: 829–836. 15244307 10.7863/jum.2004.23.6.829

[32] Ying M, Ahuja AT (2006) Ultrasound of neck lymph nodes: How to do it and how do they look? Radiography 12: 105–117.

[33] Zenk J, Bozzato A, Steinhart H, Greess H, Iro H (2005) Metastatic and inflammatory cervical lymph nodes as analyzed by contrast-enhanced color-coded doppler ultrasonography: Quantitative dynamic perfusion patterns and histopathologic correlation. Ann Otol Rhinol Laryngol 114: 43. 15697161 10.1177/000348940511400108

[34] Van den Broeck W, Derore A, Simoens P (2006) Anatomy and nomenclature of murine lymph nodes: Descriptive study and nomenclatory standardization in BALB/cAnNCrl mice. J Immunol Methods 312: 12–19. 16624319 10.1016/j.jim.2006.01.022

[35] Bosisio MR, Maisonneuve C, Gregoire S, Kettaneh A, Mueller CG, et al (2009) Ultrasound biomicroscopy: A powerful tool probing murine lymph node size *in vivo* . Ultrasound Med Biol 35: 1209–1216. 19427105 10.1016/j.ultrasmedbio.2009.02.005

[36] Mancini M, Vergara E, Salvatore G, Greco A, Troncone G, et al (2009) Morphological ultrasound microimaging of thyroid in living mice. Endocrinology 150: 4810–4815. 19589864 10.1210/en.2009-0417

[37] Charles R, Iezza G, Amendola E, Dankort D, McMahon M (2011) Mutationally activated BRAFV600E elicits papillary thyroid cancer in the adult mouse. Cancer Res 71: 3863–3871. 21512141 10.1158/0008-5472.CAN-10-4463 PMC3107361

[38] Diallo-Krou E, Yu J, Colby LA, Inoki K, Wilkinson JE, et al (2009) Paired box gene 8-peroxisome proliferator-activated receptor-γ fusion protein and loss of phosphatase and tensin homolog synergistically cause thyroid hyperplasia in transgenic mice. Endocrinology 150: 5181–5190. 19797117 10.1210/en.2009-0701 PMC2775974

[39] Roths JB, Murphy ED, Eicher E (1984) A new mutation, gld, that produces lymphoproliferation and autoimmunity in C3H/HeJ mice. J Exp Med 159: 1–20. 6693832 10.1084/jem.159.1.1 PMC2187205

[40] Takahashi T, Tanaka M, Brannan CI, Jenkins NA, Copeland NG, et al (1994) Generalized lymphoproliferative disease in mice, caused by a point mutation in the fas ligand. Cell 76: 969–976. 7511063 10.1016/0092-8674(94)90375-1

[41] Pezold JC, Zinn K, Talbert MA, Desmond R, Rosenthal EL (2006) Validation of ultrasonography to evaluate murine orthotopic oral cavity tumors. ORL 68: 159–163. 16465070 10.1159/000091324

[42] Ehlers S, Hölscher C, Scheu S, Tertilt C, Hehlgans T, et al (2003) The lymphotoxin β receptor is critically involved in controlling infections with the intracellular pathogens mycobacterium tuberculosis and listeria monocytogenes. The Journal of Immunology 170: 5210–5218. 12734369 10.4049/jimmunol.170.10.5210

[43] Alyas F, Lewis K, Williams M, Moody A, Wong K, et al (2005) Diseases of the submandibular gland as demonstrated using high resolution ultrasound. Br J Radiol 78: 362–369. 15774602 10.1259/bjr/93120352

[44] Li L, Mori S, Kodama M, Sakamoto M, Takahashi S, et al. (2013) Enhanced sonographic imaging to diagnose lymph node metastasis: Importance of blood vessel volume and density. Cancer Res. 10.1158/0008-5472.CAN-12-4200 23333937

[45] Li L, Mori S, Sakamoto M, Takahashi S, Kodama T (2013) Mouse model of lymph node metastasis via afferent lymphatic vessels for development of imaging modalities. PloS one 8: e55797. 23405215 10.1371/journal.pone.0055797 PMC3565997

[46] Sapino A, Cassoni P, Zanon E, Fraire F, Croce S, et al (2003) Ultrasonographically-guided fine-needle aspiration of axillary lymph nodes: Role in breast cancer management. Br J Cancer 88: 702–706. 12618878 10.1038/sj.bjc.6600744 PMC2376348

[47] Zagorianakou P, Fiaccavento S, Zagorianakou N, Makrydimas G, Stefanou D, et al (2005) FNAC: Its role, limitations and perspective in the preoperative diagnosis of breast cancer. Eur J Gynaecol Oncol 26: 143–149. 15857017

[48] van Herpen CM, van der Laak, Jeroen AWM, de Vries, I Jolanda M, van Krieken JH, de Wilde PC, et al (2005) Intratumoral recombinant human interleukin-12 administration in head and neck squamous cell carcinoma patients modifies locoregional lymph node architecture and induces natural killer cell infiltration in the primary tumor. Clinical cancer research 11: 1899–1909. 15756016 10.1158/1078-0432.CCR-04-1524

[49] Woolgar JA, Triantafyllou A, Lewis Jr JS, Hunt J, Williams MD, et al. (2012) Prognostic biological features in neck dissection specimens. European Archives of Oto-Rhino-Laryngology: 1–12. 10.1007/s00405-012-2170-9 22983222

[50] Qian C, Berghuis B, Tsarfaty G, Bruch M, Kort EJ, et al (2006) Preparing the “soil”: The primary tumor induces vasculature reorganization in the sentinel lymph node before the arrival of metastatic cancer cells. Cancer Res 66: 10365–10376. 17062557 10.1158/0008-5472.CAN-06-2977

[51] Lee SY, Chao-Nan Q, Seng OA, Peiyi C, Bernice WHM, et al (2012) Changes in specialized blood vessels in lymph nodes and their role in cancer metastasis. Journal of Translational Medicine 10: 206. 23035663 10.1186/1479-5876-10-206 PMC3551724

[52] Chung MK, Jung E (2012) Lymphatic vessels and high endothelial venules are increased in the sentinel lymph nodes of patients with oral squamous cell carcinoma before the arrival of tumor cells. Annals of surgical oncology 19: 1595–1601. 22124758 10.1245/s10434-011-2154-9

