# Supplementary figures and images for: Use of High Frequency Ultrasound to Monitor Cervical Lymph Node Alterations in Mice
Source: PLoS One. 2014 Jun 23;9(6):e100185. doi: 10.1371/journal.pone.0100185 (PMC4067293; doi:10.1371/journal.pone.0100185)

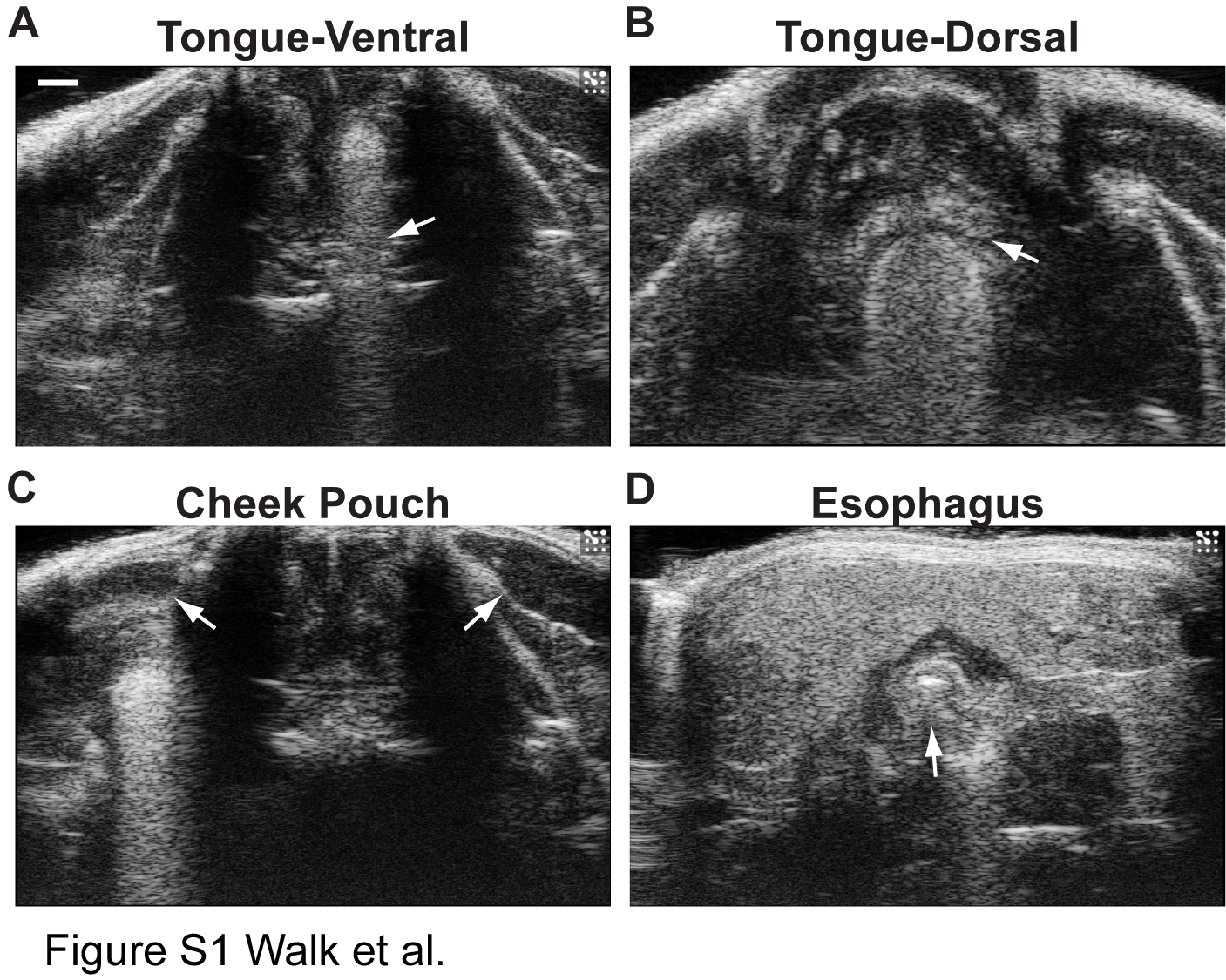

Supplement: Figure S1 — Visualization of regions within the mouse neck by high-frequency ultrasound. HF US imaging of the mouse oral cavity following placement of an oral gavage needle in mouth to dampen the US signal, aiding with identification of different sections during imaging. Arrows point to area probed with needle, which can be seen in each image blocking ultrasound signal. Scale bar = 1 mm. (TIF) [file pone.0100185.s001.tif]

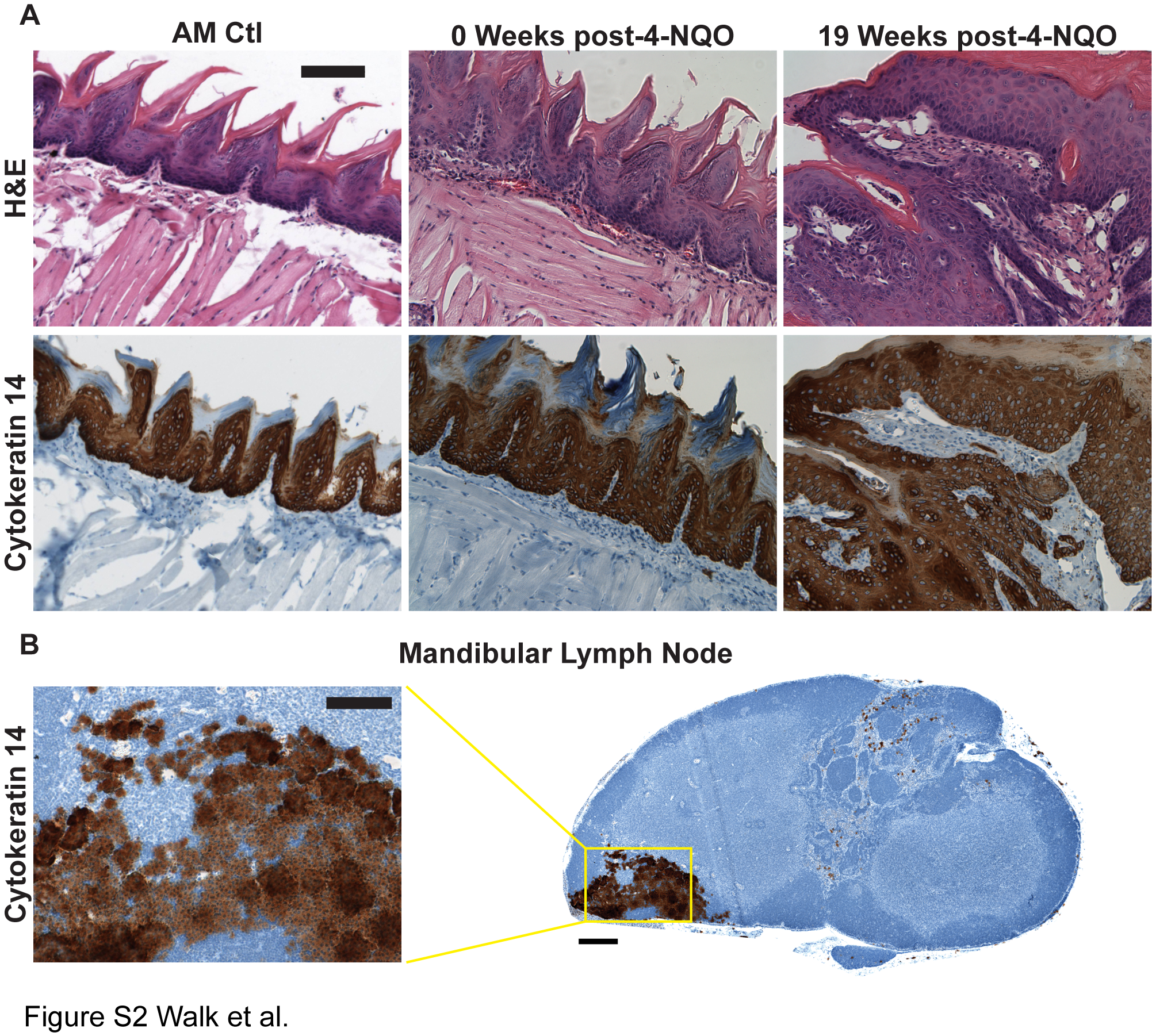

Supplement: Figure S2 — 4-NQO exposure induces changes in mouse tongue epithelium similar to human HNSCC and results in cervical lymph node metastasis. A. H&E and cytokeratin 14 staining of representative mouse tongues: control untreated, after 8 weeks of treatment, and after termination at 19 weeks due to tumor burden as an example to validate the ability of 4-NQO to induce oral tumors. Scale bar = 100 µm. B. Cytokeratin 14 staining of mouse mandibular node from 4-NQO-treated animal 33 weeks after the end of 4-NQO treatment. Inset demonstrates cytokeratin 14-positive cells indicating epithelial origin, confirming tumor metastasis. (TIF) [file pone.0100185.s002.tif]
